# Mindfulness Meditation Improves Musical Aesthetic Emotion Processing in Young Adults

**DOI:** 10.3390/ijerph182413045

**Published:** 2021-12-10

**Authors:** Xiaolin Liu, Huijuan Shi, Yong Liu, Hong Yuan, Maoping Zheng

**Affiliations:** 1 Key Laboratory of Cognition and Personality, Ministry of Education, Southwest University, Chongqing 400715, China; Liumusicpsy@163.com (X.L.); liuy0768@swu.edu.cn (Y.L.); yuanyh@swu.edu.cn (H.Y.); 2 School of Music, Southwest University, Chongqing 400715, China; 3 School of Music, Chongqing Institute of Foreign Studies, Chongqing 401120, China; shishi1984219@sina.com; 4 School of Psychology, Southwest University, Chongqing 400715, China

**Keywords:** mindfulness meditation, musical aesthetics, aesthetic emotion, executive control, ERPs

## Abstract

This study explored the behavioral and neural correlates of mindfulness meditation improvement in musical aesthetic emotion processing (MAEP) in young adults, using the revised across-modal priming paradigm. Sixty-two participants were selected from 652 college students who assessed their mindfulness traits using the Mindful Attention Awareness Scale (MAAS). According to the 27% ratio of the high and low total scores, participants were divided into two subgroups: high trait group (*n* = 31) and low trait group (*n* = 31). Participants underwent facial recognition and emotional arousal tasks while listening to music, and simultaneously recorded event-related potentials (ERPs). The N400, P3, and late positive component (LPC) were investigated. The behavioral results showed that mindfulness meditation improved executive control abilities in emotional face processing and effectively regulated the emotional arousal of repeated listening to familiar music among young adults. These improvements were associated with positive changes in key neural signatures of facial recognition (smaller P3 and larger LPC effects) and emotional arousal (smaller N400 and larger LPC effects). Our results show that P3, N400, and LPC are important neural markers for the improvement of executive control and regulating emotional arousal in musical aesthetic emotion processing, providing new evidence for exploring attention training and emotional processing. We revised the affecting priming paradigm and E-prime 3.0 procedure to fulfill the simultaneous measurement of music listening and experimental tasks and provide a new experimental paradigm to simultaneously detect the behavioral and neural correlates of mindfulness-based musical aesthetic processing.

## 1. Introduction

Enjoying music is a favored artistic experience for human beings, and musical aesthetic processing profoundly impacts human life [1]. Researchers have determined that, just as with the needs for food and sex, the aesthetic significance of music lies in the pleasant experiences and reward values induced by music exposure [2,3,4,5,6]. Previous studies have revealed that emotions are the core features of people enjoying music [1,2,7,8]. When individuals are immersed in music, focusing on cognitive-emotional interpretation and judgment, listening to music usually brings an aesthetic experience [9,10,11] and induces positive aesthetic emotions [1,4,7,12,13]. Compared with discrete emotions (e.g., happiness, sadness, calm, fear, love-warmth), music induces more delicate and complex aesthetic emotions, such as nostalgia, transcendence, curiosity, strength, etc. [2,7,14,15,16]. In the study of musical aesthetic emotion processing (MAEP) [5], the aesthetic emotion model [1,7,12,15,17] may be more suitable than the traditional discrete emotion model [18,19] and the two-dimensional emotion model [20]. Aesthetic emotion theory belongs to a specific field of emotion modeling [1,7,12,15], and is the theoretical basis for this study.

Previous studies have achieved rich results from exploring aesthetic emotion processing in music, confirming that in a safe aesthetic context, listening to music can regulate emotions, induce positive and pleasant experiences, produce reward effects, promote physical and mental health, and enhance well-being in adolescents as well as young and old adults [2,4,21,22,23]. However, previous studies mostly explored the behavioral and neural mechanisms of music-induced emotions and aesthetic experience from the perspective of music ontology, and paid less attention to the influence of individual attentional, physical, and mental states on the processing of musical aesthetic emotions [24,25]. There is a lack of psychological evidence that explores whether temporary mindfulness meditation can improve the behavioral and neural correlation of MAEP among individuals with different trait mindfulness [5].

As a training method for improving sustained attention and self-control, mindfulness meditation can effectively promote physical and mental relaxation, induce attention to the present, and enhance attention processing [26,27,28,29]. Mindfulness meditation is a self-regulation method in which individuals consciously sustain their attention on the internal or external experience without judging it in the present moment [30,31]. Scientific research on mindfulness meditation has regarded it as a personality trait possessed by individuals and an attention state cultivated through mindfulness meditation training [31,32], which is divided into trait mindfulness and state mindfulness [29,31]. Trait mindfulness can be measured using a variety of validated self-report questionnaires, such as the Mindful Attention Awareness Scale (MAAS) [33,34]. State mindfulness could be measured using the Toronto Mindfulness Scale (TMS) [35], and refers to the cultivation of individual mindfulness meditation attentional awareness through mindfulness meditation training, which in turn induces attention in the present moment, psychological and physical relaxation, and non-judgment [29,31]. Previous studies demonstrated that state and trait mindfulness effectively promote emotional processing and enhance emotional regulation [31,36,37]. Neurophysiological evidence suggests that mindfulness meditation leads to positive changes in brain structure and function that are involved in attentional processes, emotion regulation, opinion-taking, and self-referential processing [38,39,40,41]. 

Previous studies have illuminated that trait mindfulness has a significant impact on individuals’ emotional processing [29]. Bailey et al. (2019) [42] used the Go/No-go task to examine the neural basis of trait mindfulness to enhance attention processing. The ERP results showed that the attention maintenance of meditation practitioners increased the P3 amplitudes at frontal sites, which is the brain region responsible for advanced cognitive processing. The activation of these brain areas is significantly related to the attention function enhancement of mindfulness meditators [42]. Brown et al. (2012) [32] used electroencephalography (EEG) to evaluate the relationship between the late positive component (LPC), also referred to as the late positive response (LPR) [43] or late positive potential (LPP) [44] and trait mindfulness among college students. The results showed that a reduction in LPC amplitude was significantly associated with high trait mindfulness. Additionally, functional magnetic resonance imaging (fMRI) has shown that an enhancement of frontal activation and a decrease in amygdala activation in emotional processing are significantly associated with state mindfulness training and trait mindfulness [29,32].

Additionally, mindfulness meditation training can effectively improve individuals’ attentional levels in emotional processing, and attention control is closely related to executive control, which includes the three core components of response control, conflict control, and inhibitory control [45]. Cross-modal affective priming is a classic experimental paradigm used to detect and evaluate executive control, as measured by EEG in emotion processing [46,47,48]. Previous studies have shown that N400 is an ERP indicator that reflects the processing of emotional meaning, and LPC (or LPR or LPP) is an ERP indicator that reflects emotional arousal. Participants’ consistent or inconsistent judgment of auditory priming and visual target stimuli induced the effect of N400 and LPC, being different expressions of the same concept. Compared with the consistent condition, the inconsistent conditions of the priming and target stimuli induced larger N400 and LPC effects [46,47,48].

Although previous studies [5,27,49] have found that mindfulness meditation has a positive effect on emotional processing and regulation of negative emotions, the impact of individuals with different trait mindfulness on the neural markers of the emotional–cognitive interaction is unclear; also, researchers have not examined whether the ERPs associated with repeated listening to familiar Chinese folk instrumental works differ among individuals with different mindfulness traits. Given that the different temporal stages of cognitive processing are affected by the induced emotional states [27], it is plausible that underlying neurophysiological differences are also present between these subgroups. To evaluate this premise, different emotional–cognitive interaction effects before and after mindfulness meditation training on the MAEP and ERPs were assessed among the high trait group (HTG) and low trait group (LTG). A revised affecting priming paradigm [46,50] was used to measure and evaluate the difference between facial recognition and emotional arousal before and after mindfulness meditation training among the subgroups. As previous ERP studies of executive control and attentional distribution emphasized the P3 and LPC components [27,48,51,52,53,54], we also adopted these ERPs as the focus of group and music with different emotional-level comparisons. Based on the assumption that coping with sustained attention induced by mindfulness meditation can positively impact emotional–cognitive interaction, we hypothesized that post-test phases would elicit smaller P3 and greater LPC amplitudes than pre-test phases in completing facial recognition and emotional arousal tasks. The current study aimed to determine the behavioral and neural correlates of listening to familiar music based on mindfulness meditation to improve MAEP. The revised facial recognition and emotional arousal tasks were used to explore the attentional distribution and executive control during music listening before and after mindfulness meditation training. The within-subject (pre- and post-test in facial recognition and emotional arousal) and between-subjects (HTG and LTG) differences in ERPs were investigated to illustrate the neural mechanisms underlying the attentional distribution and executive control of repeated listening to familiar music. Based on previous studies, we hypothesized the following:

- First, compared with the pre-test, the post-test of facial recognition would have a higher accuracy (ACC) and faster response times (RTs), which would be reflected in smaller P3 and larger LPC amplitudes at the post-test.
- Second, compared with the LTG, there would be higher ACC and faster RTs in facial recognition, which would be reflected in that the smaller P3 and larger LPC amplitudes in the HTG. There would be no significant difference between the groups in the post-test of facial recognition.
- Third, compared with the pre-test, the post-test of emotional arousal would have a lower intensity and faster RTs, which would be reflected in smaller N400 and larger LPC amplitudes at the post-test.
- Fourth, compared with the LTG, there would be lower intensity and faster RTs in emotion arousal, which would be reflected in smaller N400 and larger LPC amplitudes in the HTG. There would be no significant difference between the groups in the post-test of emotional arousal.
- Fifth, in terms of music aesthetic emotions, the intensity of the post-test would be lower than that of the pre-test; the emotional intensity for the HTG would be higher than that for the LTG.

## 2. Methods

### 2.1. Participants

Sixty-two participants (71.67% female, M = 20.35, SD = 2.05) were selected from 652 college students who assessed their mindfulness traits using the MAAS [33,34]. According to the 27% ratio of the high and low total MAAS scores, participants were divided into two subgroups: the HTG (*n* = 31) and the LTG (*n* = 31). All participants were required to abstain from taking substances or medications that could potentially influence their concentration. They reported having normal speech and hearing, normal or corrected-to-normal vision, and being right-handed. Additionally, none of them reported having any history of major psychological disorders or receiving training related to mindfulness meditation. The study was approved by the Southwest University Ethics Committee (IRB No. H19072).

### 2.2. Stimuli

#### 2.2.1. Musical Stimuli

The stimuli set consisted of six complete Chinese classical folk instrumental works, which were taken from the commercially available “Kugou” music software (see Liu et al., 2021a [27] and Liu et al., 2021b [5]). These music stimuli included three emotion levels (calm, happy, and sad) and the duration of each was approximately 3 min. Fifty musicians used a 9-factorial GEMS model [15,17] to assess the emotional valence of the musical materials. The Cronbach’s alpha values for the calm, happy, and sad music were 0.87, 0.82, and 0.89, respectively. The participants reported that they were familiar with all musical stimuli. The emotional content of these musical materials were calm music (M = 81.24, SD = 11.85), happy music (M = 82.63, SD = 12.18), and sad music (M = 79.32, SD = 17.61).

In this stimuli set, two complete musical works with the same emotional level formed a sub-experiment task; a total of three subtasks are included as calm, happy, and sad music. To eliminate the influence of familiarity with the music on the participants’ musical aesthetic emotional processing, all participants were required to listen to these six pieces of music more than five times a day for seven days before the experiment. Participants reported all the music was familiar, and no significant difference between the groups in familiarity with the music was observed (the HTG, M = 6.37, SD = 1.32; the LTG, M = 6.41, SD = 0.98).

#### 2.2.2. Mindfulness Meditation Audio

The Chinese version of the mindfulness meditation script [5,27] was chosen for this study. The duration of the audio recording was 10 min. 

### 2.3. Self-Reported Measures

#### 2.3.1. The Positive and Negative Affect Schedule

Participants’ current mood state in terms of negative and positive affect was assessed using the Positive and Negative Affect Schedule (PANAS), which is a 20-item questionnaire [55]. As originally reported, Cronbach’s alphas of the scale were as follows: positive affect (PA) ranged from 0.86 to 0.9, and those for negative affect (NA) ranged from 0.84 to 0.87 [55]. The PANAS had a Cronbach’s alpha of 0.69 in this study. Since the mood state of the participants may affect the potential effects of mindfulness meditation training, the PANAS was used is for its measurement and evaluation.

#### 2.3.2. Mindful Attention Awareness Scale

The MAAS is a questionnaire used to measure the level of awareness of individual mindfulness traits [33,34]. The Chinese version of the MAAS was revised by Chen Siyi et al. and Yu-Qin Deng et al. in 2012 [34,56], and has 15 items. Participants are required to report their actual feelings in the last week on a 6-point scale ranging from 1 “almost always” to 6 “almost never”. The higher the score, the higher the level of awareness and attention in daily life. In this study, the MAAS was used to measure and evaluate participants’ attentional awareness level of their mindfulness traits, and had a Cronbach’s alpha of 0.91.

#### 2.3.3. The Toronto Mindfulness Scale 

The TMS is a promising measure of the mindfulness state with good psychometric properties [27], and is widely used to measure state mindfulness [35,57,58]. Cronbach’s alphas for the scale were as follows: Curiosity ranged from 0.62 to 0.82, and Decentering ranged from 0.56 to 0.78. In this study, participants’ mindfulness meditation state before and after the intervention was measured using the TMS, which had a Cronbach’s alpha of 0.87.

### 2.4. Facial Recognition and Emotional Arousal Tasks

**Figure 1:**
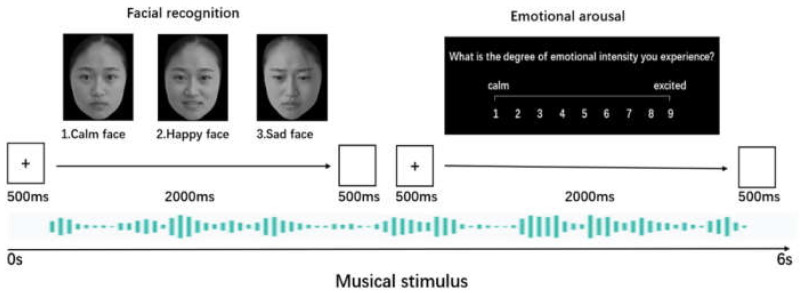
An example of the experimental task.

The facial recognition and emotional arousal tasks (Figure 1) were revised by the cross-modal affective priming paradigm [43,46,47,48,50] and were used to measure and evaluate the differences between- and within-groups in the MAEP. The priming stimuli were complete Chinese classical folk instrumental works with three emotional levels (calm, happy, and sad); target stimuli consisted of a combination of 40 pictures of the same faces with three different emotion levels (calm, happy and sad). Participants were required to assess the congruency of the priming and target stimuli as quickly as possible. These facial stimuli were selected from the “Same Face Spontaneous Multiple Expression Pool” [59]. The experimental task was divided into two subtasks (facial recognition and emotional arousal), comprising 160 trials. In the facial recognition task, the pictures with the three emotion levels in the combination are numbered 1, 2, and 3, which corresponded to the keys on the computer keyboard. A fixation point was presented for 500 ms, the three same-face pictures of the three emotional levels were presented simultaneously for 2000 ms, followed by a 500 ms blank screen. The participants were required to press the number keys (1, 2, 3) as quickly as possible. In the emotional arousal task, participants were required to report their intensity of emotional arousal through the number keys (1 “calm,” 9 “excited”), and press the number keys (1–9) as quickly as possible. The fixation point was presented for 500 ms, then the emotional arousal was presented for 2000 ms, followed by a 500 ms blank screen. To eliminate the practice effect, we created a pseudo-random combination of three pictures of the same face (calm, happy, and sad face), and performed the presentation order of the three sub-experiment tasks: calm, happy, and sad music. 

### 2.5. Procedure

After obtaining informed consent forms, the PANAS was used to rate participants’ mood state level, the MAAS was used to assess their trait mindfulness, and the TMS was used to measure their state mindfulness. All participants were asked to listen twice to music at each emotion level, and were “asked to practice mindfulness meditation using the audio material of temporary mindfulness meditation training, which took 10 min” [5] before listening to the second music piece. In the second listening session, to effectively maintain the state of mindfulness meditation, before the second and third subtasks were presented, participants practiced a brief mindfulness meditation for 2 min. Participants completed the experimental task while listening to the music and filled out the AESTHEMOS after listening to each emotion level of music. The participants’ EEG data were recorded simultaneously during the experiment. Finally, participants were asked to complete the TMS and MAAS again. 

### 2.6. Behavioral Analyses

Behavioral analysis is employed to evaluate the behavioral performance of participants in the facial recognition and emotional arousal tasks. The between-group differences in age and PANAS scores were explored by independent-samples t-tests. The between-group differences in TMS, MAAS, and AESTHEMOS scores were analyzed using repeated-measures analysis of variance (ANOVA). Repeated-measures ANOVA (2 tasks (post-test, post-test) × 3 musical emotion level (calm music, happy music, sad music) × 2 groups (high trait group, HTG; low trait group, LTG)) were conducted for the ACC and RTs of facial recognition and emotional arousal in the experimental task, with task and musical emotion level as a within-subject factor and group as a between-subjects factor. SPSS 22.0 was used to analyze the data. The Greenhouse–Geisser method was used to adjust the *p*-values for sphericity. The Bonferroni adjustments for multiple comparisons were used to perform post-hoc *t*-tests. 

### 2.7. EEG Recording and Analyses

Brain electrical activity was recorded from 32 scalp sites using tin electrodes mounted on an elastic cap (Neuroscan, Charlotte, NC, USA), with reference electrodes placed on REF (fronto-central aspect) and a ground electrode on the medial frontal aspect (GRD). The vertical electrooculogram (IO) was recorded with an electrode placed infraorbitally near the left eye. All inter-electrode impedances were maintained below 5 kΩ. Using the EEGLAB toolbox 14.1.1b, ERPs data processing was performed using MATLAB R2014a. 

The same-face stimuli and emotional arousal were created by individual’s grand ERP averages, which were based on the correct trials. The data from 1000 to 256 Hz was downsampled and high- and low-pass filtering at 0.1 Hz and 45 Hz, respectively, was performed. We selected the left and right mastoids as the reference sites. Data were epoched from 200 ms prior to stimulus onset to 1000 ms after the presentation, and were baseline-corrected to the pre-stimulus interval. Trials were excluded if they included electro-oculogram (EOG) artifacts (ocular movements and eye blinks); artifacts due to amplifier clipping, bursts of electromyographic activity, or peak-to-peak deflections exceeding ±80 μV also were excluded from averaging before independent component analysis (ICA). There were no between- or within-group differences in the trial counts for the facial recognition and emotional arousal tasks. Moreover, the components of the EOG artifacts and head movement were removed from the results of the ICA results after visual inspection. 

Based on previous studies and across-modal emotion processing of the topographical distribution of the grand-averaged ERP activities (see Liu et al., 2021b [27]), their time epochs were selected for analysis: two early time windows from 250 to 500 ms (N400 component [27,46,47,60]) and from 300 to 600 ms (P3 component, [27,54]), and a late time window from 600 to 1000 ms (late positive component: LPC, [27,43,46]). These ERP component latencies were assessed relative to the onset of the auditory stimulus, which included three levels of musical emotion (calm, happy, and sad music). The following regions of interest (ROIs) (see Liu et al., 2021b [27]) were selected [27,54,61]: frontal (F3, Fz, F4), frontal–central (FC3, FCz, FC4), central (C3, Cz, C4), central–parietal (CP3, CPz, CP4), parietal (P3, Pz, P4), and occipital (O1, OZ, O2). For the experimental task, repeated-measures ANCOVA (2 (task: post-test, post-test) × 3 (musical emotion level: calm music, happy music, sad music) × 2 (group: HTG, LTG) × 6 (ROIs: frontal, frontal–central, central, central–parietal, parietal, and occipital sites)) were conducted on the amplitudes of P3, N400, and LPC, with group as a between-subjects factor and task, musical emotion level, and ROIs as within-subject factors. SPSS 22.0 was used to analyze the data. The Greenhouse–Geisser method was used to adjust the *p*-values for sphericity. The Bonferroni adjustments for multiple comparisons were used to perform post-hoc *t*-tests. 

## 3. Results

### 3.1. Self-Reported Results

**Figure 2:**
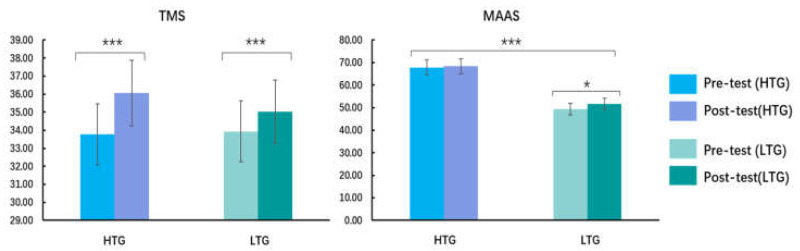
Toronto Mindfulness Scale (TMS) difference within-group and Mindful Attention Awareness Scale (MAAS) difference between-group before and after mindfulness meditation training. HTG: high trait group, LTG: low trait group; * *p <* 0.05, *** *p <* 0.001.

The participants’ demographic information and self-reported results are shown in Table 1 and Figure 2. 

No significant between-group differences in age or sex were found (all *p* > 0.05). Independent-samples *t*-tests showed that there was no between-group difference in the PANAS scores (*p* > 0.05). Repeated-measures ANOVA on TMS scores showed that there was a main effect of measure (Figure 2), *F* (1, 60) = 13.00, *p* = 0.001, $\eta_{p}^{2}$ = 0.18, and the post-hoc *t*-test displayed that the post-test scores in the TMS total scores were higher than the pre-test. No main effect of group and no interaction between measure and group were observed (all *p* > 0.05). There was a main effect of group on the MAAS scores (Figure 2), *F* (1, 60) = 85.34, *p* < 0.001, $\eta_{p}^{2}$ = 0.59, and the MAAS scores of the HTG were higher than those of the LTG. This result indicated that the trait level of mindfulness meditation in the HTG was significantly higher than that in the LTG. Additionally, mindfulness meditation training improved the levels of the mindfulness trait among individuals with a low mindfulness trait, and had no significant effect on the level of individuals with a high mindfulness trait. No main effect of measure and no interaction between measure and group were found (all *p* > 0.05). 

### 3.2. Behavioral Results

**Figure 3:**
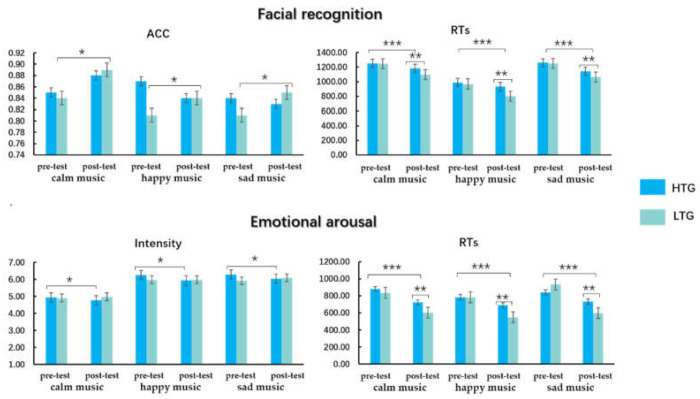
Accuracy (ACC) and reaction times (RTs) difference within-group and between-group in the facial recognition and emotional arousal task; HTG: high trait group, LTG: low trait group; * *p* < 0.05, ** *p* < 0.01, *** *p* < 0.001.

The behavioral results of the participants’ facial recognition and emotional arousal on the three levels of musical emotion are shown in Table 2 and Figure 3.

#### 3.2.1. Facial Recognition

Repeated-measures ANOVA on the ACC of facial recognition found that there was no main effect of measure, musical emotion level, or group (all *p* > 0.05). In the ACC of facial recognition, there was a marginally significant interaction effect between musical emotion level and measure (*F* (2, 60) = 3.46, *p* = 0.06, $\eta_{p}^{2}$ = 0.06). Simple effect analysis showed that the ACC of the post-test was greater than that of the pre-test in facial recognition for calm and sad music (*p* < 0.05), with no significant differences in the ACC of facial recognition for happy music. An interaction between measure and group was marginally significant, *F* (1, 60) = 3.66, *p* = 0.06, $\eta_{p}^{2}$ = 0.06, and a simple effect analysis suggested that the ACC of the post-test was greater than that of the pre-test for the LTG (*p* = 0.02); no significant difference between pre- and post- tests of the HTG was found (*p* > 0.05). There was no interaction between musical emotion level and group (all *p* > 0.05).

There was a main effect of the measure on RTs of facial recognition (*F* (1, 60) = 123.62, *p* < 0.001, $\eta_{p}^{2}$ = 0.67), and RTs of the post-test were higher than that of the pre-test. There was a main effect of musical emotion level, *F* (2, 60) = 183.01, *p* < 0.001, $\eta_{p}^{2}$ = 0.75, and the post-hoc *t*-test showed that the RTs of happy music were faster than those of calm and sad music (*p* < 0.001), with no significant differences in calm and sad music (*p* > 0.05). There was a marginally significant difference in the main effect of the group (*F* (1, 60) = 3.33, *p* < 0.07, $\eta_{p}^{2}$ = 0.05), and RTs for facial recognition for the HTG were slower than those for the LTG. An interaction between measure and group was recorded (*F* (1, 60) = 14.71, *p* < 0.001, $\eta_{p}^{2}$ = 0.20), and a simple effect analysis found that RTs for the post-test for the HTG were slower than those for the LTG (*p* = 0.002), with no significant within-group differences in pre-test RTs in facial recognition (*p* > 0.05). There was an interaction between the musical emotion level and measure, *F* (2, 60) = 2.90, *p* = 0.06, $\eta_{p}^{2}$ = 0.05, and the RTs of the post-test on facial recognition were faster than that of the pre-test on all three levels of musical emotion. The interaction effect of musical emotion level and group was not recorded (all *p* > 0.05). 

#### 3.2.2. Emotional Arousal

A repeated-measures ANOVA on emotional arousal found that a main effect of musical emotion level was recorded (*F* (2, 60) = 33.97, *p* < 0.001, $\eta_{p}^{2}$ = 0.37), and the emotional intensities of happy and sad music were greater than those of calm music (*p* < 0.001); there was no significant difference between happy and sad music (*p* > 0.05). There were no main effects of measure and group (all *ps* > 0.05). An interaction between measure and group was found, *F* (1, 60) = 4.51, *p* = 0.038, $\eta_{p}^{2}$ = 0.07. Simple effect analysis displayed that emotional arousal in the pre-test was greater than that in the post-test for the HTG (*p* = 0.027), with no significant difference between the pre- and post-test for the LTG (*p* > 0.05). There was no interaction between musical emotion level and measure, musical emotion level, or group (all *ps* > 0.05). 

The main effect of RTs on emotional arousal was observed, *F* (1, 60) = 155.40, *p* < 0.001, $\eta_{p}^{2}$ = 0.72, and the RTs in the post-test were faster than those in the pre-test for emotional arousal. The main effect of musical emotion level was recorded (*F* (2, 60) = 5.81, *p* = 0.004, $\eta_{p}^{2}$ = 0.09), and the RTs for happy music were faster than those for calm and sad music (*p* = 0.01); no significant differences were found in calm and sad music. There was no significant difference between the groups (*p* > 0.05). An interaction between measure and group was observed, *F* (1, 60) = 15.47, *p* < 0.001, $\eta_{p}^{2}$ = 0.21, and the RTs of the LTG were faster than those of the HTG in post-test emotional arousal (*p* = 0.01); there were no significant between-group differences in the RTs of the pre-test. An interaction between the musical emotion level and measure and group was found (all *p* > 0.05).

#### 3.2.3. The AESTHEMOS Results

**Figure 4:**
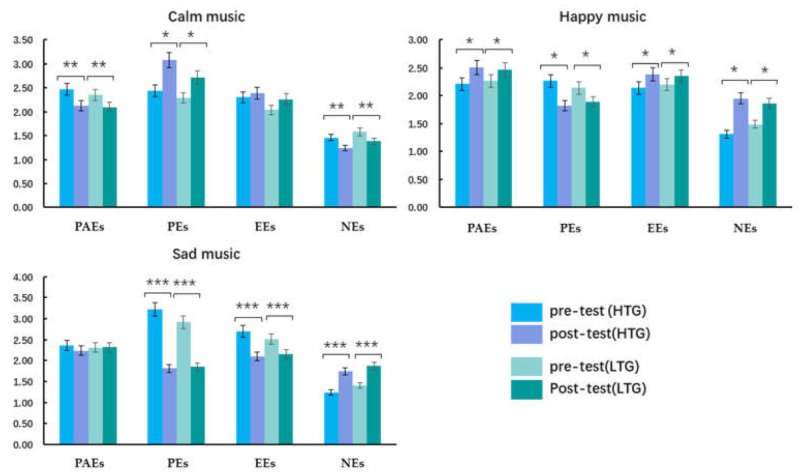
Aesthetic Emotions Scale (AETHEMOS) differences within- and between-group on the three levels of musical emotion. PAEs: prototypical aesthetic emotions, PEs: pleasing emotions, EEs: epistemic emotions, NEs: negative emotions; HTG: high trait group, LTG: low trait group; M: mean, SD: standard deviation; * *p <* 0.05, ** *p <* 0.01, *** *p* < 0.001.

The results of the participants’ aesthetic emotion experience on the three levels of musical emotion are shown in Table 3 and Figure 4.

#### 3.2.4. Calm Music

Repeated-measures ANOVA on the experience of aesthetic emotions showed no main effect of measure and group (all *ps* > 0.05). A main effect of emotion type on AESTHEMOS was observed, *F* (3, 60) = 138.58, *p* < 0.001, $\eta_{p}^{2}$ = 0.70, and there were significant differences in emotional types (pleasing emotions *>* prototypical aesthetic emotions > epistemic emotions > negative emotions). An interaction between the measure and emotion types was recorded (*F* (3, 60) = 25.08, *p* < 0.001, $\eta_{p}^{2}$ = 0.30), and the post-test score was greater than that of the pre-test on pleasing emotions (*p* < 0.05); the post-test score was lower than that of pre-test on prototypical aesthetic and negative emotions (*p* < 0.01), with no significant difference between pre- and post-test on epistemic emotions. There were no interaction effects between measure and group and emotion types and group (all *p* > 0.05). 

#### 3.2.5. Happy Music

Repeated-measures ANOVA on the experience of aesthetic emotions showed that a main effect of measure was recorded, *F* (1, 60) = 5.93, *p* = 0.018, $\eta_{p}^{2}$ = 0.09, and the experiences of the post-test on prototypical aesthetic emotions were greater than those of the pre-test. There was a main effect of emotion type (*F* (3, 60) = 43.12, *p* < 0.001, $\eta_{p}^{2}$ = 0.42), with ratings of emotional intensity in prototypical aesthetic emotions > epistemic emotions > pleasing emotions > negative emotions. No significant difference between the groups was found (*p* > 0.05). An interaction effect of emotion types and measure was found, *F* (3, 60) = 21.48, *p* < 0.001, $\eta_{p}^{2}$ = 0.26, and post-test scores on prototypical aesthetic emotions, epistemic emotions, and negative emotions were higher than those of the pre-test (*p* = 0.02); the post-test score on pleasing emotions was lower than that of the pre-test (*p* = 0.038). Interactions between the measure and group and emotion types and groups were not found (all *p* > 0.05).

#### 3.2.6. Sad Music

Repeated-measures ANOVA on the experience of aesthetic emotions found that the main effect of the measure was recorded, *F* (1, 60) = 25.32, *p* < 0.001, $\eta_{p}^{2}$ = 0.30, and the experiences of the post-test on prototypical aesthetic emotions were lower than those of the pre-test. A main effect of emotion type was observed (*F* (3, 60) = 43.12, *p* < 0.001, $\eta_{p}^{2}$ = 0.42), with ratings of emotional intensity of pleasing emotions > prototypical aesthetic emotions > epistemic emotions > negative emotions. A between-group difference was found (*p* > 0.05). An interaction effect of emotion types and measure was found (*F* (3, 60) = 75.98, *p* < 0.001, $\eta_{p}^{2}$ = 0.56), and there was no significant difference in the pre- and post-test on prototypical emotions (*p* > 0.05). The scores of the post-test on pleasing and epistemic emotions were lower than those of the pre-test (*p* < 0.001), and the post-test score on negative emotions was greater than that of the pre-test (*p* < 0.001). Interactions between measure and group and emotion types and groups were not recorded (all *p* > 0.05).

### 3.3. The ERPs Results

**Figure 5:**
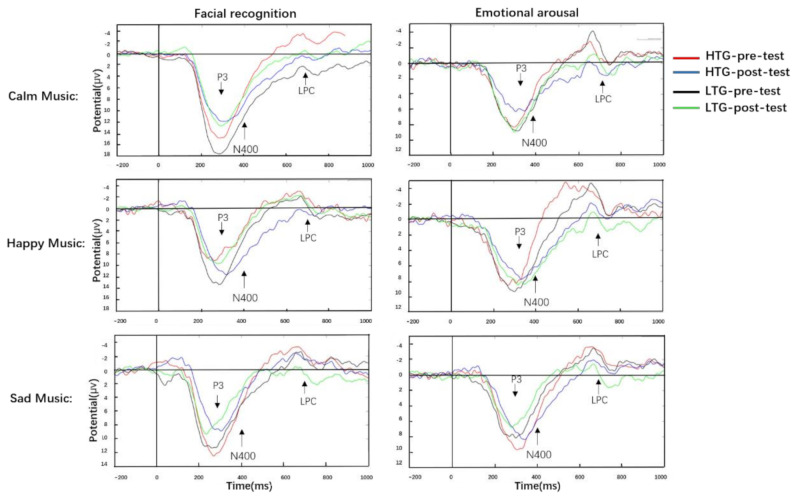
Grand average waveforms of P3 and late positive component (LPC) at site Fz in the facial recognition and emotional arousal task. HTG: high trait group; LTG: low trait group.

Grand average ERPs for the facial recognition and emotional arousal task of P3, and LPC at FCz, are shown in Figure 5.

#### 3.3.1. P3

Regarding P3, there was a main effect of measure on facial recognition (*F* (1, 60) = 11.17, *p* < 0.001, $\eta_{p}^{2}$ = 0.16), and the P3 mean amplitudes at the post-test were lower than those in the pre-test. No significant between-group difference was recorded (*p* > 0.05). There was a main effect of musical emotion level, *F* (2, 60) = 19.72, *p* < 0.001, $\eta_{p}^{2}$ = 0.25, and the post-hoc *t*-test found that P3 mean amplitudes of the calm music were greater than those of happy and sad music (*p* < 0.001); there was no difference between happy and sad music. A main effect of ROIs was observed, *F* (5, 60) = 123.63, *p* < 0.001, $\eta_{p}^{2}$ = 0.67, with the P3 mean amplitudes of ROIs in Fz *>* FCz *>* Cz *>* CPz *>* Pz *>* Oz. No interaction between measure and musical emotion level, measure and ROIs, and measure and group were found (all *p* > 0.05). 

In the emotional arousal task, there was no main effect of, and no interaction between, measure and group (all *p* > 0.05). 

#### 3.3.2. N400

In the facial recognition task, no main effect of measure and group and no interaction between measure and group were observed (all *p* > 0.05). 

In the emotional arousal task, we found a main effect of measure (*F* (1, 60) = 6.09, *p* = 0.016, $\eta_{p}^{2}$ = 0.09), and the N400 mean amplitudes at the post-test were lower than those at the pre-test. There was no main effect of group or musical emotion level (all *ps* > 0.05). There was a main effect of ROIs, *F* (5, 60) = 17.76, *p* < 0.001, $\eta_{p}^{2}$ = 0.61, with the N400 mean amplitudes of ROIs in Fz < FCz < Cz < CPz < Pz < Oz. There was an interaction between measure and group (*F* (1, 60) = 4.39, *p* = 0.04, $\eta_{p}^{2}$ = 0.07), and the N400 mean amplitudes at the post-test were lower than those at the pre-test in the HTG (*p* = 0.04), with no significant pre- and post-test differences in the LTG (*p* > 0.05). A triple interaction between measure, ROIs, and group was found, *F* (5, 60) = 4.39, *p* = 0.04, $\eta_{p}^{2}$ = 0.07, and N400 mean amplitudes of the post-test at FCz, Cz, and CPz were lower than those of the pre-test in the HTG (*p* < 0.05); there was no significant difference between pre- and post-test in the LTG (*p* > 0.05). There was no interaction between the measure and musical emotion level or measure and group (all *p* > 0.05). 

#### 3.3.3. LPC 

In the facial recognition task, there was a main effect of measure (*F* (1, 60) = 2.92, *p* = 0.07, $\eta_{p}^{2}$ = 0.05), and the LPC mean amplitudes at the post-test were higher than those at the pre-test. We also found a main effect of ROIs (*F* (5, 60) = 27.85, *p* < 0.001, $\eta_{p}^{2}$ = 0.32), and the LPC mean amplitudes of the six ROIs were significantly different, greatest from high to low: Fz *>* FCz *>* Cz *>* CPz *>* Pz *>* Oz. No main effect of group or musical emotion levels were found (all *p* > 0.05). There were no interactions between measure and group, measure and ROIs, and measure and musical emotion level (all *p* > 0.05). However, a quadruple interaction was observed, *F* (10, 60) = 2.56, *p* = 0.005, $\eta_{p}^{2}$ = 0.04. Simple effect analysis showed that the LPC amplitudes at Fz and FCz for the HTG were significantly smaller than those for the LTG in the pre-test facial recognition of calm music (*p* < 0.05). There were no significant differences between-group in the rest of ROIs and in the post-test facial recognition (all *ps* > 0.05). In the pre- and post-test facial recognition of happy music, there was no significant difference between groups in the LPC amplitude of all ROIs (all *p* > 0.05). In the pre-test facial recognition of sad music, the LPC amplitudes at Fz for the HTG were significantly smaller than those for the LTG (*p* = 0.06), with no significant differences between groups in the rest of the ROIs and in all ROIs of the post-test facial recognition (all *p* > 0.05). 

In the emotional arousal task, we found a main effect of measure (*F* (1, 60) = 16.05, *p* < 0.001, $\eta_{p}^{2}$ = 0.21), and the LPC mean amplitudes at the post-test were higher than those at the pre-test. The main effect of the musical emotion level was recorded (*F* (2, 60) = 4.05, *p* = 0.02, $\eta_{p}^{2}$ = 0.06), and the P3 mean amplitudes of calm music were greater than those of happy and sad music (*p* < 0.05), with no difference between calm and sad and happy and sad music. We also found a main effect of ROIs (*F* (5, 60) = 7.81, *p* < 0.001, $\eta_{p}^{2}$ = 0.12), and the LPC mean amplitudes of ROIs at Fz were the greatest, with no significant differences among the five ROIs (FCz, Cz, CPz, Pz, and Oz). There was no significant difference between the groups (*p* > 0.05). An interaction between the measure and ROIs (*F* (1, 60) = 16.05, *p* < 0.001, $\eta_{p}^{2}$ = 0.21) was observed, and the LPC mean amplitudes of the post-test at Fz, FCz, Cz, CPz, and Pz were greater than those of the pre-test (*p* < 0.05); there was no difference between the pre- and post-test at Oz. There were no interactions between the measure and musical emotion level, and the measure and group and musical emotion level and group were not recorded (all *ps* > 0.05).

## 4. Discussion

In our novel examination of the behavioral and ERP correlates of mindfulness meditation improving MAEP, the hypotheses were partially confirmed. The findings show that (1) in the facial recognition of three levels of musical emotion, the ACC of the post-test is higher than that of the pre-test for the LTG, and there was no significant difference in the pre- and post-test for the HTG; however, the RTs of the post-test are faster than that of the pre-test for the HTG and the LTG. ERP results indicate that P3 is an effective ERP index for facial recognition in emotional–cognitive interactions. The smaller P3 amplitude reflects that the processing of emotional faces in the state of mindfulness meditation consumes the less advanced cognitive resources. (2) In the emotional arousal of the three levels of musical emotion, arousal at the post-test was greater than that of the pre-test for the HTG, with no significant difference between pre- and post-test for the LTG; RTs of the post-test were faster than that of the pre-test on RTs of emotional arousal for the HTG and LTG, RTs of the LTG are faster than that of the HTG at the post-test, and there was no difference between groups at pre-test. N400 is an effective ERP indicator for detecting emotional arousal processing. The reduction in the N400 amplitudes at central–frontal, central, and central–parietal sites indicates that mindfulness meditation reduces the arousal intensity of individuals with high trait mindfulness and sustains the arousal intensity of individuals with low trait mindfulness. LPC is an effective ERP component for maintaining attentional processing and inducing emotions. The higher LPC amplitudes reflect that mindfulness meditation enhances individuals’ attentional maintenance and emotional arousal. Additionally, the AESTHEMOS results show that mindfulness meditation also effectively regulates the musical aesthetic emotional experience of individuals with high and low trait mindfulness. In the emotional experiences of calm music, mindfulness meditation enhances the pleasing and epistemic experiences, and reduces the prototypical aesthetic and negative experiences. In the emotional experiences of happy music, mindfulness meditation improves the prototypical aesthetic and epistemic experiences, reduces pleasure experiences, and increases the negative experiences. In the emotional experiences of sad music, mindfulness meditation maintains the prototypical aesthetic experiences, and reduces pleasing, epistemic, and negative experiences.

In the facial recognition task, mindfulness meditation improved the emotional face processing of individuals with high and low trait mindfulness. In our revised same-face recognition task, participants were asked to assess the faces that were consistent with the emotional valence of music as soon as possible while listening to music. Behavioral results showed that in the facial recognition, mindfulness meditation improved the reaction times of all participants and the accuracy of the LTG. This indicates that mindfulness meditation promotes executive control of emotional facial processing [28]. From the perspective of emotional valence, the emotional state induced by music with different emotional levels also significantly affects the executive control ability of individuals with mindfulness traits, and the individual’s executive function is related to the positive emotional state induced by stimuli [27,54]. Our results show that happy faces were responded to significantly faster than calm and sad faces, indicating that positive mood states can effectively improve individual executive functions [27,31,62,63]. It is worth noting that there was no significant difference between groups at pre-test RTs in facial recognition, and RTs for the LTG were faster than those for the HTG at post-test. This suggests that mindfulness meditation can effectively improve the executive control ability of individuals with low trait mindfulness. 

Our ERP results suggest that P3 and LPC are effective EEG components that reflect the processing of emotional faces, and the decrease in P3 amplitude is related to individual emotional induction [27,54], and the increase in LPC amplitude reflects the need to consume more attention resources [51,52,53]. In the facial recognition task, compared with the pre-test, the same-face processing at the post-test induced smaller P3 mean amplitudes. Furthermore, the P3 amplitudes of calm music in the pre- and post-test tasks of facial recognition were significantly larger than those of happy and sad music, while there was no significant difference between happy and sad music. This result is consistent with previous studies [27,53,64], which found that, compared with neutral emotional states, positive or negative emotional states will induce a smaller P3 amplitude [27,53,54]. Previous studies have shown that LPC is related to attention distribution and emotional integration [43,48]. The change in LPC amplitude depends on the allocation of cognitive resources; that is, the more cognitive resources the task requires, the greater the LPC amplitude [65]. The increase in LPC amplitude reflects the need to consume more attentional resources in stimulus processing [27,51,52,53], which is closely related to individual motivation or emotional induction [43,44]. Our results show that the participants’ behavioral performance dovetailed with their ERP results. In the pre-test task of facial recognition of calm and sad music, the LPC amplitudes for the LPC at Fz and FCz were significantly smaller than those for the LTG, with no significant differences between groups at the post-test task of face recognition in all levels of music emotion. This result indicates that in the late processing of the pre-test task on facial recognition, individuals with low trait mindfulness consumed more advanced cognitive resources than individuals with high trait mindfulness. However, in the post-test task of face recognition, mindfulness meditation more effectively promoted the same-face processing of individuals with low trait mindfulness, and improved their ability for executive control [27,31].

A previous study has shown that repeatedly listening to familiar music significantly reduces emotional arousal and musical exposure, while unfamiliar music can effectively maintain individual listening interest [66]. Our results suggest that in the emotional arousal task, mindfulness meditation regulated the emotional arousal of listening repeatedly to familiar music. Mindfulness meditation reduces the emotional arousal of individuals with high trait mindfulness and maintains the emotional arousal of individuals with low trait mindfulness. In the executive control of emotional arousal, RTs of the post-test were faster than those of the pre-test. This shows that mindfulness meditation not only improves the executive control ability of individuals with high or low trait mindfulness but also effectively regulates the intensity of emotional arousal. This provides new evidence to explain the listening repeatedly to familiar music. Our ERP results were consistent with the behavioral performance of emotional arousal. In the emotional arousal task, larger LPC amplitudes reflected that the individuals who completed the experimental tasks needed to consume more attention resources under the state of attention maintenance and emotion induction. Previous studies have found that LPC is an ERP index that reflects emotional arousal [53] and attentional maintenance [26]. The change in LPC amplitude is closely related to an individual’s motivation or emotional induction [66]. The increase in LPC amplitude indicates that individuals need to consume more attentional resources under emotional induction and attentional maintenance [27,44,46,61]. The behavioral and ERP results in the emotional arousal task show that mindfulness meditation can effectively regulate the emotional processing of individuals with high or low trait mindfulness. Most notably, in the performance of facial recognition and emotional arousal tasks, mindfulness meditation can more effectively promote executive control ability and emotional arousal in individuals with low trait mindfulness.

In an aesthetic context, individuals tend to listen to music that is consistent with their mood [67]. The AESTHEMOS results indicated that mindfulness meditation effectively regulated the effects of repeated listening to familiar music. In the aesthetic emotional experience of calm music, mindfulness meditation enhanced the pleasant and epistemic emotional experiences of individuals with high and low trait mindfulness and reduced the aesthetic and negative experiences. In the aesthetic emotional experience of happy music, mindfulness meditation enhanced their pleasant and epistemic emotional experiences, and reduced the aesthetic and negative emotional experiences. In the aesthetic emotional experience of sad music, mindfulness meditation maintained the aesthetic experience, reduced the pleasure and epistemic experiences, and increased the negative experiences. These results show that mindfulness meditation significantly affected an individual’s enjoyment of music and aesthetic experience, improving the individual’s MAEP [5]. 

Some limitations of this study should be acknowledged. Although MAEP improved by mindfulness meditation is relevant and common among young adults, the findings of this study may not be generalizable to other populations, e.g., younger or older age groups or non-Chinese participants. These populations should be focused on in future studies. Furthermore, although our results reflect possible neural remarks in response to mindfulness-based repeated listening to music, with distinct emotional valences improving MAEP, it is not clear whether the pattern of effects would extend to liking-based music and Chinese vocal music works. Additionally, in the future, fMRI should be used to explore the neural basis of short-term mindfulness meditation on music aesthetic emotion processing, and to reveal the role of mindfulness music listening in improving the perceptual experience and promoting physical and mental health. Finally, the study introduces a new direction for future research employing mindfulness-based music therapy to treat mental disorders in community mental health centers.

## 5. Conclusions

In summary, mindfulness meditation significantly improves the executive control ability of individuals with high and low trait mindfulness on emotional face processing and effectively regulates the aesthetic emotional experience of repeatedly listening to familiar music. The behavioral performance in facial recognition and emotional arousal tasks and the EEG characteristics of P3, N400, and LPC provide new evidence for people to deeply understand the inner mechanism of mindfulness meditation on the aesthetic emotion processing of individuals with high or low trait mindfulness. Our research results show that the facial processing and emotional arousal of young adults is closely related to the following ERP components: P3 is related to the consistent cognitive evaluation of emotional facial stimuli and is an effective ERP indicator for detecting emotional face processing. The decrease in P3 amplitudes indicates that mindfulness meditation improves the executive control of young adults in the processing of facial recognition. N400 is an effective ERP indicator for detecting emotional arousal processing; the reduction in N400 amplitudes at the central–frontal, central, and central–parietal sites indicates that mindfulness meditation reduces the arousal intensity of individuals with high trait mindfulness and sustains the arousal intensity of individuals with low trait mindfulness. LPC is an ERP index that effectively detects attention maintenance and emotional arousal during emotional processing. When repeatedly listening to familiar music, the individuals’ mindfulness meditation maintenance and emotional arousal induced greater LPC amplitudes at the frontal sites. Our results show that P3, N400, and LPC are important EEG components in facial recognition and emotional processing, providing new evidence for exploring attention training and emotional processing.

## Figures and Tables

**Table 1:** Participants’ demographic information and self-reported results.

Variables		HTG (M ± SD)	LTG (M ± SD)	t
		*n* = 31	*n* = 31	
Age		20.45 (2.46)	20.03 (1.47)	0.81
Sex		Male = 9, female = 21	Male = 6, female = 25	
PANAS	PA	2.55 (0.77)	2.32 (0.77)	1.29
	NA	1.24 (0.34)	1.48 (0.54)	1.06
TMS ***	pre-test	33.77 (5.59)	33.94 (3.95)	0.13
	post-test	36.06 (5.13)	35.03 (4.69)	0.83
MAAS ***	pre-test	67.84 (6.08)	49.35 (6.62)	11.45
	post-test	68.35 (8.33)	51.61 (11.11)	6.71

Note: Positive and Negative Affect Schedule (PANAS) difference within-group at the mood state after experiment; Toronto Mindfulness Scale (TMS) and Mindful Attention Awareness Scale (MAAS) difference within- and between-group before and after mindfulness meditation training; PA: positive affect, NA: negative affect; HTG: high trait group, LTG: low trait group; M: mean, SD: standard deviation; *** *p* < 0.001.

**Table 2:** Descriptive statistics of the facial recognition and emotional arousal task.

Variables		HTG (M ± SD)			LTG (M ± SD)		
		*n* = 31			*n* = 31		
MELs		CM	HM	SM	CM	HM	SM *
FR	ACC (pre-test)	0.85 (0.13)	0.87 (0.16)	0.84 (0.14)	0.84 (0.13) *	0.81 (0.20) *	0.81 (0.15) *
	ACC (post-test)	0.88 (0.16)	0.84 (0.27)	0.83 (0.20)	0.89 (0.08) *	0.84 (0.22) *	0.85 (0.14) *
	RTs (pre-test)	1249.35 (98.53)	990.24 (146.33)	1260.40 (140.16)	1244.06 (180.83)	969.95 (161.59)	1249.87 (178.15)
	RTs (post-test) ***	1182.51 (120.15)	933.15 (159.44)	1142.08 (113.97)	1098.00 (162.02)	804.39 (174.47)	1063.99 (165.88)
EE	Arousal (pre-test)	4.94 (1.49) *	6.26 (1.06) *	6.29 (1.44) *	4.91 (1.18)	5.98 (1.19)	5.93 (1.27)
	Arousal (post-test)	4.78 (0.68) *	5.94 (1.24) *	6.04 (1.45) *	4.98 (1.24)	5.98 (1.28)	6.10 (1.55)
	RTs (pre-test)	879.71 (217.31)	783.92 (193.73)	839.47 (225.83)	834.12 (244.99)	781.94 (249.18)	930.89 (270.46)
	RTs (post-test) **	723.24 (226.59)	687.23 (232.43)	731.09 (222.66)	604.07 (197.26)	550.67 (186.84)	597.28 (231.15)

Note: MELs: musical emotion levels; FR: facial recognition, EA: emotional arousal; ACC: accuracy, RTs: reaction times; HTG: high trait group, LTG: low trait group; CM: calm music, HM: happy music, SM: sad music; M: mean, SD: standard deviation; * *p <* 0.05, ** *p <* 0.01, *** *p* < 0.001.

**Table 3:** Descriptive statistics of AESTHEMOS results.

Variables	HTG (M ± SD)			LTG (M ± SD)		
	*n* = 31			*n* = 31		
MELs	CM	HM *	SM	CM	HM *	SM
PAEs (pre-test)	2.47 (0.74) *	2.21 (0.78)	2.36 (0.71)	2.35 (0.57)	2.27 (0.66)	2.31 (0.60)
PAEs (post-test)	2.13 (0.67) *	2.51 (0.83)	2.24 (0.81)	2.10 (0.51)	2.47 (0.65)	2.32 (0.74)
PEs (pre-test)	2.44 (0.66) **	2.27 (0.81)	3.22 (0.81) ***	2.29 (0.67)	2.14 (0.61)	2.92 (0.72) ***
PEs (post-test)	3.08 (0.92) **	1.82 (0.68)	1.81 (0.54) ***	2.72 (0.82)	1.89 (0.74)	1.85 (0.54) ***
EEs (pre-test)	2.30 (0.69)	2.14 (0.83)	2.70 (0.75) ***	2.04 (0.56)	2.20 (0.69)	2.51 (0.61) ***
EEs (post-test)	2.39 (0.78)	2.38 (0.69)	2.10 (0.64) ***	2.26 (0.62)	2.35 (0.69)	2.16 (0.73) ***
NEs (pre-test)	1.46 (0.48) **	1.31 (0.36)	1.24 (0.33) ***	1.58 (0.42)	1.49 (0.58)	1.41 (0.54) ***
NEs (post-test)	1.24 (0.38) **	1.95 (0.66)	1.74 (0.46) ***	1.38 (0.47)	1.86 (0.54)	1.87 (0.51) ***

Note: AETHEMOS: Aesthetic Emotions Scale; MELs: musical emotion levels; CM: calm music, HM: happy music, SM: sad music; PAEs: prototypical aesthetic emotions, PEs: pleasing emotions, EEs: epistemic emotions, NEs: negative emotions; HTG: high trait group, LTG: low trait group; M: mean, SD: standard deviation; * *p <* 0.05, ** *p <* 0.01, *** *p* < 0.001.
